# A systematic review on the causes of the transmission and control measures of outbreaks in long-term care facilities: Back to basics of infection control

**DOI:** 10.1371/journal.pone.0229911

**Published:** 2020-03-10

**Authors:** Min Hye Lee, Gyeoung Ah Lee, Seong Hyeon Lee, Yeon-Hwan Park

**Affiliations:** 1 The Research Institute of Nursing Science, College of Nursing, Seoul National University, Seoul, South Korea; 2 College of Nursing, Seoul National University, Seoul, South Korea; University of Nottingham, UNITED KINGDOM

## Abstract

**Background:**

The unique characteristics of long-term care facilities (LTCFs) including host factors and living conditions contribute to the spread of contagious pathogens. Control measures are essential to interrupt the transmission and to manage outbreaks effectively.

**Aim:**

The aim of this systematic review was to verify the causes and problems contributing to transmission and to identify control measures during outbreaks in LTCFs.

**Methods:**

Four electronic databases were searched for articles published from 2007 to 2018. Articles written in English reporting outbreaks in LTCFs were included. The quality of the studies was assessed using the risk-of-bias assessment tool for nonrandomized studies.

**Findings:**

A total of 37 studies were included in the qualitative synthesis. The most commonly reported single pathogen was influenza virus, followed by group A streptococcus (GAS). Of the studies that identified the cause, about half of them noted outbreaks transmitted via person-to-person. Suboptimal infection control practice including inadequate decontamination and poor hand hygiene was the most frequently raised issue propagating transmission. Especially, lapses in specific care procedures were linked with outbreaks of GAS and hepatitis B and C viruses. About 60% of the included studies reported affected cases among staff, but only a few studies implemented work restriction during outbreaks.

**Conclusions:**

This review indicates that the violation of basic infection control practice could be a major role in introducing and facilitating the spread of contagious diseases in LTCFs. It shows the need to promote compliance with basic practices of infection control to prevent outbreaks in LTCFs.

## Introduction

Outbreak of an infectious disease is defined as the occurrence of a disease above the expected level [1]. Over the past several years, many countries have experienced serious economic and health consequences due to outbreaks of infectious diseases such as the Middle East Respiratory Syndrome in 2015 and Severe Acute Respiratory Syndrome in 2003.

Long-term care facilities (LTCFs) are facing a great need for preparation for infection outbreaks because of an increase in the number of residents with global aging. LTCFs are exposed to the risk of outbreaks owing to several factors. First, older residents in LTCFs are susceptible to infectious diseases because of aging and health conditions [2] and are known to be dependent with regard to activities of daily living. Thus, among residents, self-hygiene is observed to be poor. Loss of independence in residents creates unique and frequent contact opportunities between healthcare workers (HCWs) and residents [3]. Second, HCWs in LTCFs tend to be poorly informed about infection prevention and control (IPC), and compliance with IPC is generally low [2, 4]. Third, the environment in LTCFs offers challenges for IPC, like the sharing of rooms, group living, and difficulty with the isolation of infected persons [5, 6]. Finally, LTCFs have limited resources and capacities for diagnosis of infection [7]. This leads to a delay in the detection of hidden carriers and infection. All these factors contribute to the onset and spread of outbreaks in LTCFs. Outbreaks in LTCFs threaten the life and health of both residents and HCWs, and thus, eliminating the risk of outbreaks is a matter of concern in such facilities. However, LTCFs vary in their individual capacities to respond to outbreaks [8]. The keys to outbreak control are as follows: identification of the transmission causes and minimization of the spread through early initiation of control measures. Therefore, it is essential to understand the causes of transmission and the applied measures to control outbreaks in LTCFs.

There are several gaps and limitations in previous studies to comprehensively understand outbreaks in LTCFs. First, in many previous researches that addressed outbreaks, the focus was on pathogens, burdens, and adverse outcomes such as mortality [9, 10]. Based on the perceived importance of control measures, one of the purposes of this review was to explore and analyze in detail the control measures reported in studies. Secondly, pharmaceutical measures may have some limitations on effectiveness against newly emerging infectious diseases or resistant strains [3] and some pathogens may not have pharmaceutical interventions to be considered during outbreaks. Non-pharmaceutical interventions (NPIs) such as hand hygiene and precautions should be utilized to prevent the transmission of outbreak pathogens, regardless of the evolution of infectious diseases. However, little attention has been paid to NPIs in studies concerning outbreaks in LTCFs [3]. This review focused on NPIs and ascertains the control measures based on the guideline for prevention and control of influenza outbreaks in LTCFs of the World Health Organization (WHO) [11]. Finally, to our knowledge, there is no published systematic review addressing overall outbreaks in LTCFs in the past 5 years.

### Objective

This review aimed to update the understanding of causes and practical issues contributing to the spread and to identify control measures during outbreaks in LTCFs, thereby improving the practice and management of outbreaks in LTCFs.

## Methods

We reported this review following the Preferred Reporting Items for Systematic Reviews and Meta-analyses (PRISMA) statement [12].

### Research questions

1. What were the sources of spread of outbreaks in LTCFs?
2. What measures were implemented to control outbreaks in LTCFs?

### PICOS statement

Population (setting/place): older adults residing or persons working in LTCFs such as a nursing home and skilled nursing facility.

Exposure (Intervention): infectious disease outbreak.

Comparison: no restriction.

Outcomes: no restriction.

Study design: outbreak investigation.

### Search strategy

The following electronic databases were searched to identify articles reporting outbreaks of infectious diseases in LTCFs using terms with Boolean operators during November 2019 (Table 1): PubMed, Excerpta Medica Database (EMBASE), Cochrane CENTRAL, and Cumulative index for nursing and allied health literature (CINAHL). Studies were limited to articles written in English language and published from 2007 to 2018 to update the understanding of outbreaks in LTCFs with more recently publication since a previous review and guideline for IPC in LTCFs were published [2, 9]. Bibliographies in studies were hand-searched.

**Table 1:** Search terms for database searches.

Database	Search terms
PubMed	((((infection[Title/Abstract] OR infections[Title/Abstract] OR outbreak*[Title/Abstract] OR transmission[Title/Abstract])) AND (“nursing home*”[Title/Abstract] OR “skilled nursing*”[Title/Abstract] OR “long-term care”[Title/Abstract])) AND (control*[Title/Abstract] OR outcome*[Title/Abstract] OR factor*[Title/Abstract])) NOT (surgery[Title/Abstract] OR cancer[Title/Abstract] OR “neoplasm”[Title/Abstract] OR “intensive care unit”[Title/Abstract] OR child[Title/Abstract] OR children[Title/Abstract] OR “operative”[Title/Abstract])
EMBASE	(‘infection’:ab,ti OR ‘infections’:ab,ti OR ‘outbreak*’:ab,ti OR ‘transmission’:ab,ti) AND (‘nursing home*’:ab,ti OR ‘skilled nursing*’:ab,ti OR ‘nursing home patient’:ab,ti) AND (‘control*’:ab,ti OR ‘outcome*’:ab,ti OR ‘factor*’:ab,ti) NOT (‘surgery’:ab,ti OR ‘cancer’:ab,ti OR ‘neoplasm’:ab,ti OR ‘intensive care unit’:ab,ti OR ‘child’:ab,ti OR ‘children’:ab,ti OR ‘operative’:ab,ti)
CINAHL	TI (infection OR infections OR outbreak* OR transmission) AND TI (“nursing home*” OR “skilled nursing*” OR “long-term care”) AND TI (control* OR outcome* OR factor*) NOT TI (surgery OR cancer OR “neoplasm” or “intensive care unit” OR child OR children OR “operative”)
	AB (infection OR infections OR outbreak* OR transmission) AND AB (“nursing home*” OR “skilled nursing*” OR “long-term care”) AND AB (control* OR outcome* OR factor*) NOT AB (surgery OR cancer OR “neoplasm” or “intensive care unit” OR child OR children OR “operative”)
Cochrane CENTRAL	infection OR infections OR outbreak* OR transmission in Title, Abstract, Keywords and "nursing home*" OR "skilled nursing*" OR "long-term care" in Title, Abstract, Keywords and control* OR outcome* OR factor* in Title, Abstract, Keywords not surgery OR cancer OR "neoplasm" or "intensive care unit" OR child OR children OR "operative" in Title, Abstract, Keywords, Publication Year from 2007 to 2016 in Trials

EMBASE, Excerpta Medica Database; CINAHL, Cumulative index for nursing and allied health literature.

### Eligibility criteria

#### Inclusion criteria

Studies (2007–2018) published in English that investigated outbreaks of any pathogen in LTCFs were considered for inclusion.

#### Exclusion criteria

Studies focusing on genetic strain of pathogens or pharmacological aspect were excluded. Surveillance reports, community outbreaks, review articles, conference papers (with unavailable full-text), and randomized controlled trials were excluded.

### Study selection

Two reviewers independently assessed the eligibility of searched studies. The titles and abstracts were primarily screened to identify whether the criteria were met. The full texts of selected studies during primary screening were reviewed for the final study selection. Any discrepancies were resolved by sharing opinions and consultation with the other author, if necessary.

### Data extraction

After pilot data extraction, two reviewers independently extracted data such as information on participants, pathogens, case definitions, number of cases and non-affected persons, overall attack rate, causes and problems that led to transmission, and control measures.

#### Control measures

General control measures considered were the formation of outbreak control team, active surveillance, standard precautions, transmission-based precautions, training and education, employee work restriction, environmental control, containment measures, and prophylaxis based on the WHO guideline [11].

### Quality assessment

The quality of studies was assessed using Risk of Bias Assessment tool for Nonrandomized Study (RoBANS) by two reviewers [13]. RoBANS is an evaluation tool for the risk of bias of non-randomized studies, with moderate reliability and acceptable validity and compatible with domains of the Cochrane risk-of-bias tool [13]. Six domains were evaluated including the selection of participants, confounding variables, exposure measurement, blinding for outcome assessment, incomplete outcome, and selective outcome reporting. According to the instruction for evaluation [13], the risk of bias for each domain was determined as low risk, unclear risk and high risk. Studies with full-text including case-control analysis, cohort study was involved in the quality assessment. Studies that simply described the results of investigation without comparative analysis were not able to evaluate the domains of the tool. Thus, the quality evaluation was not conducted with this type of study. Any difference was discussed between the two reviewers, and, if necessary, an agreement was reached with the corresponding author. The result of quality assessment was displayed using Review Manager (RevMan) version 5.3 software (The Cochrane Collaboration, Oxford, UK).

## Results

### Search results

**Fig 1:**
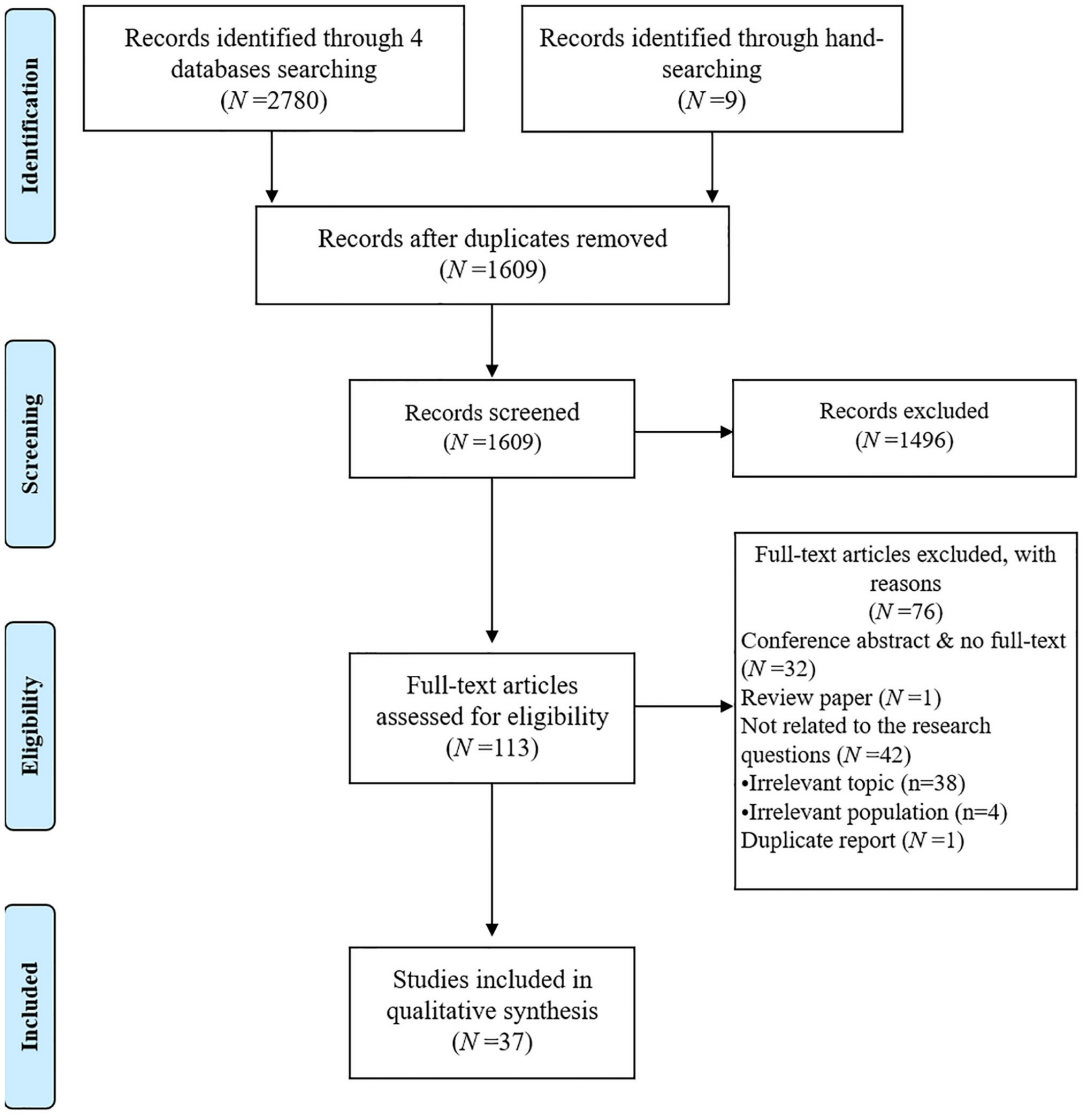
PRISMA flow diagram of the study selection [12].

A total of 2,789 studies were retrieved from 4 databases and hand searched. The duplicate records were removed (n = 1180), and the eligibility criteria were applied for the selection process. After reviewing the full text, 76 articles were excluded for the following reasons: irrelevant for the research topic (n = 38) and population (n = 4), unavailable full-text (n = 32), review article (n = 1), and duplicated report (n = 1). Finally, 37 articles were included in this review (Fig 1).

### Characteristics of the included studies

Characteristics of the eligible studies are presented in Table 2. Over half of the included studies (n = 22) were published since 2013. The majority of the included studies were reported in the United States (n = 15) and Europe (n = 13) followed by Asia (n = 5).

**Table 2:** Characteristics of the included studies and the outbreaks (N = 37).

Characteristics	N or N (%)
**Publication year**	
2007–2008	6(16.22)
2009–2010	4(10.81)
2011–2012	5(13.51)
2013–2014	6(16.22)
2015–2016	11(29.73)
2017–2018	4(10.81)
2019*	1(2.70)
**Location**	
US	15(40.54)
Europe	13(35.14)
Australia	1(2.70)
Canada	3(8.11)
Asia	5(13.51)
**Outbreak pathogen**	
** Bacterial**	
Multi-drug resistant organisms	3(8.11)
Group A Streptococcus	5(13.51)
* Clostridium difficile*	1(2.70)
* Salmonella enteritidis*	1(2.70)
* Clostridium perfringens*	1(2.70)
* Haemophilus influenzae*	2(5.41)
* Mycobacterium tuberculosis*	2(5.41)
** Viral**	
Influenza viruses	6(16.22)
Hepatitis B virus	4(10.81)
Hepatitis E virus	1(2.70)
Hepatitis C virus	1(2.70)
Rotavirus	1(2.70)
Norovirus	3(8.11)
Adenovirus	1(2.70)
Multiple	5(13.51)
**Duration of Outbreaks**	
< 1 month	13(35.14)
1–6 months	10(27.03)
> 6 months	13(35.14)
Not reported	1(2.70)
**Causes of transmission**^†^	
Person-to-person transmission	14(35.90)
Problems in practice	8(20.51)
Contaminated water and food	5(12.82)
Not identified or not reported	12(30.77)
**Critical problems in practice related to outbreaks**^†^	
Hand hygiene	11
Use of personal protective equipment	6
Cleaning and disinfection	8
Sharing of devices	3
Inappropriate use of reusable devices	1
Environmental infection control (e.g. room renovation, ventilation)	4
Delayed notification of outbreak	2
Timing of implementation of control measures	4
Delayed diagnosis of infection and recognition of outbreaks	4
Issues related to vaccine	3
Work restriction for ill employee	3
Personal hygiene of staff members	1
Limited application of isolation and cohorting	1
Lack of targeted training for practitioner	1
Lack of communication between institutions	1
Understaffing	1

US, The United States.

*E-pub in 2018

^†^multiple count

### Risk of bias in the included studies

**Fig 2:**
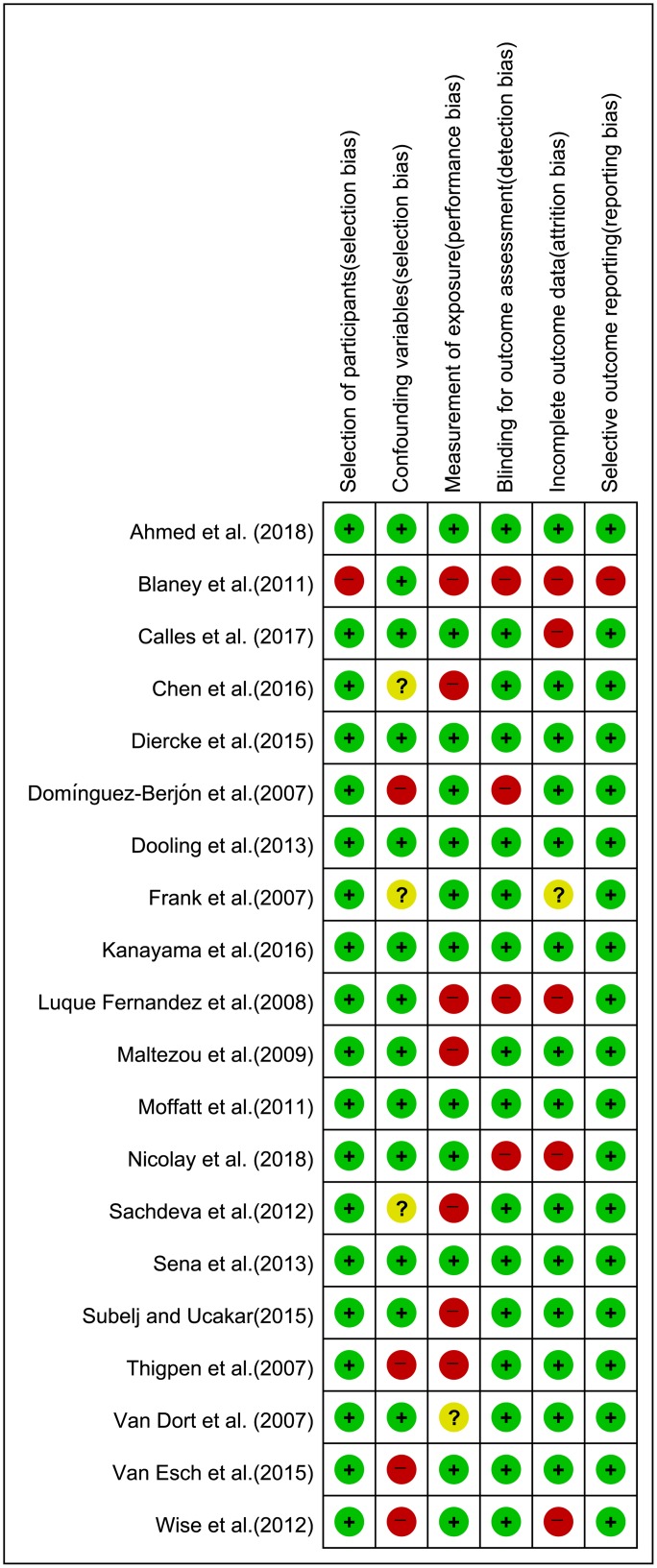
Risk of bias summary (Revman 5.3). indicating low (+), unclear (?), and high (-) risk of bias.

The quality of 20 studies was assessed and the results are summarized in Fig 2. One study was at high risk for five criteria [14]. Six studies were at low risk for all criteria [15–20]. Problems related to recall bias and standardization of self-reported measurement created a high risk of bias for the measurement of the exposure domain in seven studies [14, 21–26]. Lack of consideration for confounders led to a high risk of bias in four studies [24, 27–29]. The problem related to missing data resulted in a high risk of bias for the incomplete outcome domain in five studies [14, 26, 28, 30, 31].

### Characteristics of the outbreaks

Characteristics of the outbreaks are presented in Tables 2–4. Fifteen studies reported outbreaks caused by bacteria [15–17, 20, 22, 24, 27, 32–39] and 22 studies were outbreaks by viruses [14, 18, 19, 21, 23, 25, 26, 28–31, 40–50]. The largest number of a single pathogen was influenza viruses [40–45], followed by group A streptococcus (GAS) [17, 20, 24, 37, 38]. The most affected site was respiratory tract (n = 12) [32, 35, 36, 39–45, 48, 50], followed by gastrointestinal (GI) tract (n = 10) [14, 16, 23, 26, 27, 31, 33, 46, 47, 49]. Other sites including skin and soft tissue and eyes were affected. The majority of the eligible studies reported one outbreak involving one facility (n = 31), while the study by Nguyen and Middaugh [49] described a gastroenteritis outbreak that was transmitted to eight facilities. Three studies analyzed the data of multiple outbreaks of viral gastroenteritis and influenza-like illness that occurred in multiple facilities for a certain period of time [14, 46, 48]. The outbreaks in 23 studies affected both the residents and HCWs [14, 20, 22, 23, 25, 26, 29, 31, 33, 35–41, 43–49], and the outbreaks of 14 studies affected only the residents [15–19, 21, 24, 27, 28, 30, 32, 34, 42, 50].

**Table 3:** Attack rate of outbreaks in the included studies*.

Etiology	Residents		Staff members		Overall	
	Median attack rate (Range)	No. of reports	Median attack rate (Range)	No. of reports	Median attack rate (Range)	No. of reports
**Respiratory tract**						
Active tuberculosis	(2.05–12.90)	2	(0.83–5.56)	2	(1.50–11.25)	2
Influenza-like illness	32.32 (9.44–51.61)	6	10.25 (0.00–19.23)	6	24.50 (9.05–42.05)	6
Respiratory syncytial virus (RSV) and human metapneumovirus (HMPV) infection	(73.17)	1	.	0	(73.17)	1
Non-typeable *Haemophilus influenzae* colonization	(72.73)	1	(12.24)	1	(23.33)	1
**Gastrointestinal tract**						
Acute gastroenteritis (norovirus, rotavirus, and *Clostridium difficile)*	41.66 (14.73–57.65)	6	19.15 (3.77–36.00)	5	32.93 (11.21–51.97)	6
*Clostridium perfringens*	(41.27)	1	.	0	(41.27)	1
*Salmonella enteritidis*	(11.44)	1	(5.15)	1	(9.64)	1
**Others**						
Group A Streptococcus (GAS) infection	1.20 (0.65–6.90)	3	(1.41–3.70)	2	2.43 (0.84–6.90)	3
Hepatitis B infection	7.82 (5.74–8.62)	4	(0.00)	1	6.78 (3.65–7.83)	4
Hepatitis C infection	(15.63)	1	(0.00)	1	(10.54)	1
Hepatitis E infection	(17.84)	1	(16.67)	1	(17.70)	1
Methicillin-resistant *Staphylococcus aureus* (MRSA)	(0.44)	1	(3.83)	1	(2.06)	1
Epidemic keratoconjunctivitis (Adenovirus)	(30.51)	1	(8.26)	1	(19.25)	1

If only one study of the outbreaks was reported, the attack rate of the study was displayed. If the number of reports was 2, only the range of attack rate was displayed.

*Blaney et al. (2011), Barret et al. (2014), Dooling et al. (2013), Nanduri et al. (2019), Mahmud et al. (2013), Kanayama et al. (2016), Weterings et al. (2015), and Van Dort et al. (2007) were excluded in this table because they were unable to calculate attack rates.

**Table 4:** Summary of the outbreaks in the included studies.

Article	Design	Pathogen or disease	Participant	Case definition	N cases/non-cases	Overall attack rate	Duration of outbreak	Transmission causes	Other problems	Control measure	Results
Šubelj and Učakar (2015) -Slovenia	Case-control study	Gastroenteritis (Rotavirus)	Residents and staffs in a LTCF	Person residing or working in the LTCF with diarrhea (≥ 3 times within 24 hrs) and at least one of the symptoms (fever, nausea, vomiting, malaise, headache and abdominal pain)	• Total: 37 cases • Case-control study: 33 cases/66 controls	11.21% Resident: 14.73% staff: 3.77%	April 11–23, 2013	Person to person transmission (delayed implementation of timely control measures)	NR	• Strict hand washing and use of PPE • Additional information on cleaning and disinfection. • Work restriction of affected employees • Restriction of movement between unit	Being ambulant (aOR: 12.3; 95%CI 1.14–133.1) and having more than two comorbidities (aOR: 4.7; 95% CI 1.1–19.2) were risk factors to acute gastrointestinal infection.
Moffatt et al. (2011)—Austral.ia	Retrospective cohort study	*Clostridium perfringens*	Residents in a 130-bed LTCF	• Possible case: resident with one or more acute loose stool episodes in 23–27 July, 2009 • Laboratory confirmed case: resident with loose stools and identified with *Clostridium perfringens* enterotoxin positive	52 cases/74 non-cases	41.3%	23–27 July, 2009	Foodborne cause	NR	NR	Cases were more likely to be male (aRR: 2.22; 95% CI 0.98–4.99, *p* = .05) and reside in Wings B (aRR: 3.26; 95% CI 1.09–9.70, *p* = .03) or C (aRR: 3.41; 95% CI 1.16–10.07, *p* = .03).
Frank et al. (2007)—Germany	Cohort study	*Salmonella enteritidis*	Residents and staffs in a nursing home with 822 residents	Persons with diarrhea and/or vomiting on any day between 31 July and 4 September and with *S*. *enteritidis* positive in stool sample	Total cases: 94 residents and 17 staff	9.64% Resident: 11.44% staff: 5.15%	24days (August, 2006)	Contaminated bakery cake (residents) and spread from case-residents to staff related close contact (staff)	NR	NR	Contaminated afternoon cake on all three days was identified as potential factors for outbreak.
Blaney et al. (2011)—New England	Outbreak analysis with comparison among facilities	Norovirus	Residents and staffs in 61 LTCFs reporting outbreaks	• AGE outbreak: illness in 2 or more residents or staff with gastroenteritis (diarrhea with ≥3 loose stools in 24 hrs, with or without vomiting) in transmission-possible period	Case/control: 27 facilities/35 facilities	43.55% (facility level)	NR	NR	NR	NR	Facilities where use of ABHS equally or more often than soap with water for hand hygiene had high chances of AGE outbreak than those with workers less likely to use ABHS (aOR: 6.06; 95% CI 1.44–33.99, *p* = .02).
Van Esch et al. (2015)—Belgium	Retrospective and prospective outbreak analysis	*Clostridium difficile*	Residents in a 120-bed LTCF	Persons with diarrhea, a positive toxin/antigen test and a positive stool culture	66 cases/61 controls	51.97%	January. 2009- December, 2012	NR	Nutritional status of residents	• Stringent hygienic protocol • Active surveillance • Strict isolation • Timely treatment for CDI (antibiotic prescription) • Cleaning and disinfection of resident rooms	The nutritional status was found to be significantly poorer in the residents with CDI.
Luque Fernandez et al. (2008)—Spain	Retrospective cohort study	Viral gastroenteritis (Norovirus and Rotavirus)	Residents and staff in a nursing home with 96 residents	Persons working or residing in the nursing home during February, 2008 who had an episode of acute diarrhea (≥3 loose stools in 24 hrs) or vomiting, or 2 or more of the symptoms (fever, nausea, abdominal pain, and malaise)	71 cases/75 controls	48.63% Resident: 55.21% staff: 36.00%	4–23 February, 2008	An infected employee of the nursing home and the tap water	NR	• Enteric isolation • Cleaning of environment and water cistern • Restriction of visitors	Persons who drank tap water had high risk of acute gastroenteritis with RR of 4.03 (95%CI, 1.4–11.4).
Barret et al. (2014)—France	Descriptive analysis of multiple outbreaks	Gastroenteritis (Norovirus 73%, Rotavirus 19%, etc)	Residents and staff in 1040 facilities	• Acute gastroenteritis: the sudden onset of diarrhea or at least two episodes of vomiting within 24 hours. • Outbreak: at least five cases of gastroenteritis within four days among residents or staff	26,551 episodes of illness among residents and 5,548 episodes of illness among staff	Mean attack rate Resident: 32.5% staff: 12.4%	November, 2010-May, 2012	Person-to-person (95%), foodborne (2%), foodborne and person-to-person (1%), waterborne (<1%)	**Problems in the management** understaffing (54%) organizational problems (45%) shortage of materials (12%) financial problems (6%)	• Reinforcement of hand hygiene (95%) • Contact precautions (87%) • Cleaning or disinfection of the environment (86%) • Restriction of movements (84%) • Stopping or limitation of group activities (58%) • Measures on food handling (44%) • Exclusion of symptomatic staff (64%)	The attack rate was lower and the duration of outbreaks was shorter when infection control measures were implemented within three days of onset of the first case.
Ludwig et al. (2013)—US	Outbreak investigation	Acute gastroenteritis (Norovirus, *Clostridium difficile*)	Residents and staff in a 120-bed LTCF	Acute gastroenteritis: ≥1 episode of vomiting or diarrhea (≥2 loose stools within 12 hours)	Cases: 30 residents and 29 staff	19.54% Resident: 15.38% staff: 27.10%	February—March, 2012	Person to person transmission	• Shared food and water • Limited enforcement of isolation and cohorting • Inadequate environmental cleaning	NR	Four cases had co-infection with *C*. *difficile* and Norovirus. Transmissions occurred probably from staff or visitors to residents.
Nguyen et al. (2012)—US	Outbreak investigation	Acute gastroenteritis (Norovirus)	Residents in 8 LTCFs	Case: a resident or staff who experienced at least ≥3 loose stools and/or ≥1 episodes of vomiting within 24 hours	299 residents, 95 staff	21.93% Resident: 31.34% staff: 11.27%	February—March, 2010 (median 11 days, range 5–33 days)	Person to person transmission	• Delayed recognition of the outbreak • Interaction by staff members who worked at multiple facilities	• Active surveillance • Cessation of new admission (Instruction to implement control measure including ill staff exclusion from work for 72 hrs after resolution of symptoms, handwashing with soap and water, and intensive environmental cleaning)	Staff members who were employed at multiple affected facilities may transmit disease between facilities.
Nicolay et al. (2018)—France	Retrospective cohort study	Acute gastroenteritis (Norovirus)	Residents and staff members in a nursing home with 89 residents	Gastroenteritis: a resident or a staff member who had sudden onset of diarrhea and/or vomiting (≥ two episodes within 24 hours)	29 residents and 9 staff	43.94% Resident: 57.65% staff: 19.15%	September 17-October 21, 2016	Person-to-person transmission	• Misuse of PPE • Inappropriate hand hygiene • Disinfection of environmental surfaces with an ineffective product on norovirus	• Reinforcement of personal hygiene and standard precaution • Barrier measures • Limitation of the movements of symptomatic residents • Environmental disinfection • Stopping group activities • Closure of the kitchen and outsourcing of meals • Meeting for awareness on barrier measures	More dependent residents were at higher risk of acute gastroenteritis [RR 2.1 (95% CI 1.1–4.1)].
Jordan et al. (2015)—US	Outbreak report	Influenza A	Residents in a skilled nursing facility	The onset of fever or respiratory illness in a resident or staff member	50 cases (44 residents and 6 staff members)	29.41% Resident: 46.32% Staff: 8.00%	November 29-December 21, 2014	NR	NR	• Prophylactic: oseltamivir • Stopping group activities and new admission • Droplet precaution • Exclusion of ill staff until 24 hours after symptom resolution	There was no significant association between illness and characteristics including age, sex, room, smoking, pneumococcal vaccination status, and chronic diseases.
Chan et al. (2014)—Hong Kong	Outbreak report	ILI (Influenza A)	Residents and staff in a nursing home	ILI: the sudden onset of any general symptoms (fever, headache, or myalgia) and respiratory symptom (cough, sore throat, or shortness of breath).	48 cases	19.59% Residents: 25.13% staff: 0.00%	July 23-August 1, 2013	NR	NR	• Prophylactic: oseltamivir • Enhance personal and environmental hygiene • Respiratory precaution • Direct observation of hand hygiene • Enhance environmental disinfection • Cohort the symptomatic residents and designated staff to care for the residents • Advise symptomatic staff to avoid work until symptoms resolution • Active surveillance • Minimize mixing activities	1. An influenza outbreak occurred in a nursing home with high vaccination rate. 2. There was no significant relationship between illness and vaccination status.
Mahmud et al. (2013)—Canada	Descriptive analysis of multiple outbreaks	Influenza A (47%) Influenza B (5%) para-influenza (5%) respiratory syncytial virus (3%) not identified (40%)	Residents and staff in 37 LTCFs	• ILI: cough and fever and one or more of sore throat, arthralgia, myalgia, and prostration. • ILI outbreak: two or more cases within 7 days	154 outbreaks	Median (influenza A and B) Resident: 7.2% staff: 3.3%	Median: 18 days (3-53days)	NR	NR	• Chemoprophylaxis: 57% of influenza A, 63% of influenza B (the other measures were not reported.)	1. Early notification to public health authorities was associated with lower attack rate and mortality rates among residents. 2. Chemoprophylaxis was the measure associated with lower attack rates, but not with shorter duration of outbreaks or with lower mortality.
Win et al. (2010)—Singapore	Outbreak investigation	ILI (Influenza B)	Residents in a 200-bed welfare home	• Probable case: fever, and either cough or running nose or sore throat, and history of contact with a confirmed case during the outbreak • Confirmed case: fever, and either cough or running nose or sore throat, and a positive test for influenza B	17 residents, 2 staff	9.05% Resident: 9.44% staff: 6.67%	16 to 21 March 2007	NR	Mismatch between the vaccine strain and the circulating strain	• Active case finding	A mismatch of vaccine can result in an outbreak in a highly immunized LTCF.
Bamberg et al. (2010)—US	Descriptive analysis of multiple outbreaks	ILI (Influenza A)	① Residents in a 39-bed LTCF ② Residents in a 125-bed LTCF ③ Residents in a 368-bed LTCF	ILI: presence of fever with cough or sore throat.	① 11 residents, 10 staff ② 7 residents, 8 staff ③ 41 residents, 135 staff	① Resident 28%, staff 40% ② Resident 6%, staff 5% ③ Resident 11%, staff 22%	October-November, 2009 ① 12–14 October ② 12–17 November ③ 26 October- 6 November	Not identified	NR	① Droplet precautions, chemoprophylaxis, Restriction of movement and visitors, vaccination ② Closing the facility to new admissions and visitors, droplet precaution, enhancement of hand hygiene and cough etiquette, ill staff exclusion from work, restriction of movement between wings, chemoprophylaxis ③ Chemoprophylaxis, enhanced surveillance, education about standard and droplet precautions, exclusion of ill staff and visitor, droplet precaution	Pandemic influenza A (H1N1) outbreaks in LTCFs in three states show that attack rates among residents varied between 6% and 28%.
Burette et al. (2009)—Belgium	Outbreak investigation	Influenza A	Residents and staff in a residence home	NR	32 residents, 5 staff	42.05% Resident: 51.61% staff: 19.23%	9–21 March, 2005	Person to person transmission (from ill staff to residents)	Mismatch between the vaccine strain and the circulating strain, 6 months after vaccination of the residents, the absence of vaccine coverage of the nursing personnel, and institutional living	• Chemoprophylaxis • Restriction within rooms for affected person,	Several factors including timing of vaccination and mismatch between the circulating strains and the vaccine strains facilitated the occurrence and spread of this outbreak.
Gaillat et al. (2008)—France	Outbreak investigation	ILI (Influenza A)	Residents and staff in a nursing home with 81residents	Case: fever >38°C combined with a cough and/or respiratory signs.	32 residents and 6 staff	29.46% Resident: 39.51% staff: 12.50%	25 June-3 July, 2005	Person-to-person transmission	Preventive heatwave measure	• Isolation • Wearing of surgical masks by all residents and staff • Droplet and contact precaution • Chemoprophylaxis • Setting up a crisis management team	This influenza outbreak occurred in the summer. The heatwave measures that all the residents were together in one limited area had an effect on the spread of the virus.
Lai et al. (2016)—Taiwan	Outbreak investigation	Tuberculosis (*Mycobacterium tuberculosis*)	Residents and staff in a 63-bed LTCF	Definite TB cases: identified by culture or molecular line probe assay and persons receiving a full course of TB treatment without diagnostic results	8 residents and 1 staff	11.25% Resident: 12.90% staff: 5.56%	September, 2011-October, 2012	Frequent movement of residents	• Insufficient fresh air exchange rate • Aerosolization of the TB patient secretions from repeated suctioning	• Active case finding screening • Contact tracing • Closing the facility to new admissions • Increasing ventilation rates • Decreasing the numbers of residents per room • Isolation and transfer to hospital	All resident cases, except for the first-floor case, had been in contact with each other in the same room. The new TST conversion rate was 25.0% for residents.
Khalil et al. (2013)—Canada	Outbreak investigation	Tuberculosis (*Mycobacterium tuberculosis*)	Residents and staff in a 121-bed residential and LTCF	Active case: a positive result for *M*. *tuberculosis* complex in culture, a positive TST or findings indicative of TB infection on x-ray or CT. New latent TB infection: a positive TST preceded by a negative TST prior to January 1, 2010.	4 active cases (3 residents and 1 staff), 24 new latent TB infection	Active cases: overall 1.50%, resident 2.05%, staff 0.83%,	May 2010-January 2011	Person to person transmission	Close living conditions, prolonged exposure due to delayed diagnosis of active cases, and air exchange rates below published guideline	• Contact surveillance and case follow-up • Closing the facility to new admission • Stopping resident transfers	Epidemiological link was found by identifying that four active cases were infected by an identical strain.
Spires et al. (2017)—US	Outbreak investigation	Respiratory syncytial virus (RSV) and human metapneumovirus (HMPV)	Residents in a LTCF	New signs or symptoms including (1) oral temperature ≥37.8°C and (2) at least 2 of the following symptoms: cough, dyspnea, rhinorrhea, hoarseness, congestion, fatigue, and malaise.	30 residents	73.17%	16 days (January 2015)	NR	• Lack of alcohol-based hand rub containers at the convenient locations • Limited supply of PPE during the outbreak	• Cohorting • Droplet and contact precaution • Stopping group activities • Chemoprophylaxis (Oseltamivir) • Active surveillance • Encourage work restriction • Emphasis on hand hygiene and respiratory etiquette • Visitor restriction • Closure of the unit to new admissions • Supply personal bottles of alcohol-based hand rub	1. Six residents were positive for RSV, 7 for HMPV and 1 resident tested positive for influenza A. 2. All patients had been diagnosed with dementia and needed some assistance for activities of daily living.
Kanayama et al. (2016)—Japan	Outbreak investigation with case-control study	MRPA	Residents in a 225-bed LTCF	Residents in whom MRPA was detected in a sputum sample taken from January to December 2013 (positive during the first 2 days of admission were excluded)	• Total: 23 cases • Case-control study: 14 cases/28 controls	NR	January, 2013-January, 2014	• The sharing of devices and violated standard precaution • Inappropriate disinfection of device (suctioning and wound care)	NR	• Active surveillance • Infection control team composition • Contact precautions • Cohorting and using new gloves and gown • Admission restriction • Training and re-education of HCWs about standard precautions • Deep environmental cleaning • Discontinuation of sharing devices	Use of an oxygen mask (aOR: 23.0; 95% Cl 2.1–250.4) and use of a nasogastric tube (aOR: 17.1; 95% CI 2.5–117.6) were significant factors associated with MRPA infection.
Maltezou et al. (2009)—Greece	Case-control study	MRSA	HCWs in a LTCF with 228 residents	HCWs with a clinically compatible *S*. *aureus* infection (*S*. *aureus* infection: a clinical skin or soft tissue infection compatible with *S*. *aureus* infection)	• Total cases: 1 resident and 8 staff • Case-control study: 8 cases/36 controls	2.06% Resident: 0.44% staff: 3.83%	November, 2006 -December 31, 2007	Transmission between nurses	Poor personal hygiene (alcohol-based antiseptic was not available and sharing clothes among practice nurses)	• Hygiene education with written materials • Contact precautions • Prophylaxis (mupirocin) • Restriction of contact with resident (HCWs with active lesion)	Working in the specific zone and being a practice nurse were found to be significant risk factors for MRSA infection.
Weterings et al. (2015)—Netherlands	Outbreak investigation	*Klebsiella pneumoniae* carbapenemase-producing *Klebsiella pneumonia* (KPC-KP)	Residents in a 150-bed nursing home	Person infected or colonized with KPC-producing Enterobacteriaceae	4 cases	NR	July-December, 2013	Inter-institutional transmission, extensive environmental contamination, and shared device	• Inappropriate glove use and poor hand hygiene compliance • Inappropriate storage of gown • Frequent transfer of patients	• Isolation (cohorting of KPC-positive patients in separate location) • PPE • Handrub with 70% alcohol • Frequent audits of hand hygiene and direct feedback • Daily cleaning of room and disinfection with hydrogen peroxide vapor • Contact screening surveillance	Preventing transmission of MDROs is challenging in nursing homes.
Dooling et al. (2013)—US	Case-control study	Group A Streptococcus (GAS)	Residents in a skilled nursing facility	Resident with onset after January 2009 with GAS isolated from a sterile or non-sterile site	• Total: 19 residents with 24 infections • Case-control study: 18 infections/54 controls	NR	June, 2009-June, 2012	Colonization of susceptible elderly residents and continued person-to-person transmission	• Insufficient placement of hand hygiene products • Poor adherence of hand hygiene • Lack of effective communication between institutions	• Carriage survey • Contact precaution (residents not receiving antibiotic prophylaxis) • Emphasizing hygiene through reminders, education, and placement of additional alcohol-based hand rub dispensers • Adequate cleaning and disinfection of equipment • Facility-wide chemoprophylaxis	Risk factors found in multivariable analysis included having an indwelling line (OR: 5.6; 95% CI 1.2–36.4) and living on wing A (OR: 3.4; 95% CI 0.9–16.4).
Thigpen et al. (2007)—US	Outbreak investigation	Group A Streptococcus (GAS)	Residents in a 146-bed nursing home	• Definite case: residents with the isolation of GAS from a sterile site • Possible case: residents with GAS isolated from a nonsterile site or a positive for an oropharyngeal swab and at least 2 of the symptoms (fever, pulmonary symptoms, pulmonary signs, altered mental, and cellulitis requiring antibiotics)	• Definite case: 6 residents • Possible case: 4 residents	6.9%	November-December, 2003	Person-person transmission	• Understaffing the months leading up to the outbreak • Absence of a sink at nursing station and lack of waterless hand sanitizers	• Screening for GAS infection by swab • Enhanced infection control measures, including (1) to reinforce use of standard precautions, (2) to improve staff access to hand disinfectants, (3) to implement appropriate respiratory hygiene practices, and (4) influenza immunization • Chemoprophylaxis for colonized persons	Three risk factors for GAS case were identified as presence of congestive heart failure or history of myocardial infarction (RR: 5.9; 95% CI 1.8–19.2), residence on unit 2 (RR: 7.9; 95% CI 1.0–62.6), and requiring a bed bath (RR: 5.3; 95% CI 1.6–17.3).
Ahmed et al. (2018)—US	Case-control study	Group A Streptococcus (GAS)	Residents and staff members in a 228-bed skilled nursing facility	• Invasive case: illness with GAS cultured from a sterile site • Noninvasive case: illness with GAS detected from a throat or wound	Infection: 7 residents and 5 staff	0.84% Resident: 0.65% staff: 1.41%	17 July, 2015–31 March, 2016	Wound irrigation of GAS-colonized or infected residents	• Non-compliance with PPE on contact precaution • Suboptimal hand hygiene adherence (62%) • Inconsistent cleaning and disinfection of shared equipment	• Active surveillance • Contact precaution • Recommendation for use of PPE during irrigation, changing soiled diapers/linen before dressing change, and adopting a supportive sick leave policy.	Residents infected with GAS were more frequently received antimicrobial treatment (*p* = .03) and wound vacuum-assisted closure devices than controls.
Nanduri et al. (2019)—US	Outbreak investigation	Group A Streptococcus (GAS)	Residents and staff in a skilled nursing facility	• Invasive case: GAS cultured from a sterile site • Noninvasive case: signs and symptoms of GAS infection and GAS cultured from a nonsterile site or detected from the throat by a rapid antigen detection test • Recurrent case: more than one invasive or noninvasive infection in the same individual identified >1 month apart.	19 invasive and 60 noninvasive cases (50 residents and 24 staff)	NR	May 2014-August 2016 (3 clusters)	Person-to-person transmission	• Low hand hygiene compliance (14–25%) • No policy for promotion of alcohol-based hand-rub dispensers • Lack of knowledge about appropriate use of PPE • Deficient wound care practice	• Chemoprophylaxis • Active surveillance • Recommendation of health authority	Inadequate infection control and wound-care practices may lead to this prolonged GAS outbreak in a skilled nursing facility.
Kobayashi et al. (2016)—US	Cross-sectional analysis and retrospective review	Group A Streptococcus (GAS)	Residents and staff in a 190-bed skilled nursing facility	• Invasive case: GAS positive cultured from a normally sterile site • Noninvasive case: GAS positive cultured from a nonsterile site with signs and symptoms of GAS infection	2 residents and 6 staff reported being diagnosed with GAS pharyngitis and receiving treatment	2.43% Resident: 1.20% staff: 3.70%	January–March, 2015	From sick staff to residents	• Low hand hygiene compliance before resident contact (68.2%) • Lapses in wound care Ineffective implement of standardized cleaning protocol for shared wound care equipment	• Surveillance culture • Contact precaution	Sick staff members may have introduced GAS into the facility, with spread by infection control lapses
Chen et al. (2016) -China	Case-control study	Hepatitis E virus	Residents and staff in a nursing home	Serum anti-HEV IgM positive, regardless of symptoms	37 cases/172 non-cases (52 controls)	17.70% Resident: 17.84% staff: 16.67%	January 13 -March 18, 2015	Tap water contamination after heavy rain	• No medical examination or screening for HEV infection at admission • Free access to the nursing home • Septic tank in water sanitation • Lack of safe excreta disposal • Tableware without disinfection and sharing drinking glasses	• Active case finding • Isolation of cases • Improvement in drinking water standards and toilet facilities, and enhancing food safety	Cases more often washed own dishes and rinsed their mouths using tap water than the controls (*p* < .05).
Diercke et al. (2015)—Germany	Retrospective cohort study	Hepatitis B virus	All residents in a nursing home	Residents with a positive result for hepatitis B surface antigen (HBsAg) and detection of the outbreak strain	Cases/non-cases: 5/59 (12 past infections)	• 7.81% • Clinical: 9.62%	July-September, 2010	Blood glucose monitoring with reusable lancet devices	NR	• Glucose monitoring procedures using single use lancets	Exposure to blood glucose monitoring was only significant factor to HBV infection in multivariate analysis (RR: 22; 95% CI 3.0-∞, *p* = .001).
Seña et al. (2013)—US	Case-control study	Hepatitis B virus	Residents in two skilled nursing facilities	Acute hepatitis B infection: serologic result with positive for HBsAg, Anti-HBc, and IgM anti-HBc and negative for Anti-HBs	• Total: 12 acute HBV cases • Case-control study: 6 cases/24 controls (for each facility)	• 5.74% • Clinical: 7.50%	A: July, 2009-January, 2010 B: April, 2010-June, 2010	Absence of trained infection control staff and suboptimal hand hygiene practices during blood glucose monitoring and insulin injections	NR	NR	1. In facility A, no factors were significantly related to acute HBV infection. 2. In facility B, exposure to blood glucose monitoring (OR: 22.0; 95% CI 2.4–204.1) and having a hospital or ER visit history (OR: 22.0; 95% CI 2.4–204.1) were related to acute HBV infection.
Wise et al. (2012)—US	Retrospective cohort study	Hepatitis B virus	Residents in a 125-bed LTCF	Acute HBV infection: positive for IgM anti-HBc	Total: 9 acute HBV cases	• 7.83% • Clinical: 11.11%	June-December, 2008	Cross-contamination of equipment and environment with blood during podiatric procedures (Improper disinfection)	NR	• Hepatitis B vaccination • Appropriate storage of equipment • Treatment room renovation • Guidance for proper cleaning and disinfection of equipment and environmental surfaces • Provision of a dedicated glucometer to all HBV-infected residents with diabetes • Periodic assessments of infection control practices	Five of 15 residents undergoing podiatric care developed acute HBV infection (rate ratio: 4.33; 95% CI 1.18–15.92).
Sachdeva et al. (2012)—Canada	Case-control study	Hepatitis B virus	Residents and staff in a long-term care home	Resident or staff who worked or lived within the LTCF during the exposure period with serological findings with acute HBV infection	5 cases/19 controls	3.65% Resident: 8.62% staff: 0.00% Clinical: 5%	April 1-November 15, 2006 (exposure period)	Blood glucose monitoring (sharing equipment among residents and poor hand hygiene adherence) and exposure to phlebotomy	NR	• Increasing cleaning and disinfection of environmental surfaces • Assignment of individual equipment for blood glucose monitoring • Restriction of non-essential personal contact • Infection control audit • Active surveillance of residents for symptoms • Post-exposure prophylaxis • Additional training for staffs	The odds of being infected with HBV increased 25% for each exposure to blood glucose monitoring per week (OR: 1.25; 95% CI 1.01–1.55, *p* = 0.04).
Calles et al. (2017)—US	Case-control study	Hepatitis C	Residents in a 114-bed skilled nursing facility	Outbreak case: resident from January 1, 2011-September 9, 2013; who was present on September 9, 2013; who was HCV positive; and whose virus was genetically related to the outbreak strain.	all cases: 45 residents case-control: 30cases/62controls	10.54% Resident: 15.63% staff: 0.00%	January 1, 2011-September 1, 2013	Not identified	• Lapses in podiatry and point-of-care procedures (inappropriate glove use) • Inconsistent cleaning of environmental surfaces and equipment	• Surveillance test • Recommendation (test on admission, monthly test, improving care treatment room, improving access to handwashing sinks, separation of clean and dirty spaces & instruments, use of single use disposable nail clipper, adequate environment cleaning and disinfection, glove change, and use of new sterile-packaged instruments)	Podiatry care and international normalized ratio monitoring by phlebotomy were significantly associated with HCV case.
Andersson et al. (2015)—Sweden	Descriptive analysis of an outbreak	Non-typeable *Haemophilus influenzae* (NTHi)	Residents and staff in a LTCF	NR	8 residents and 6 staff	23.33% Resident: 72.73% staff: 12.24%	October, 2011	NR	NR	• No new admission • Education for hygiene and cohorting of staff • Surveillance culture for resident and staff	This was an outbreak of an NTHi with high virulence.
Van Dort et al. (2007)—US	Case-control study	*Non-typeable Haemophilus influenzae (NTHi)*	Residents in a 120-bed nursing home	Person with culture-positive non-typeable *Haemophilus influenzae* (NTHi)	13 cases/18 controls	NR	June-July, 2005 (6weeks)	NR	NR	• Universal precaution • Respiratory droplet precaution • Evaluating staffs with symptoms Throat culture survey for residents	None of the variables showed a significant association with the NTHi.
Domínguez-Berjón et al. (2007)—Spain	Cohort study	Adenovirus	Residents and staffs in a nursing home with 118 residents	Person who showed ≥3 of the signs (conjunctival redness or edema, lid edema, or lacrimal sac swelling) and ≥1 of the symptoms (eye pain, photophobia) with a clinical course longer than 48 hrs, and no other cause	46 cases (36 residents and 10 HCWs)/193 controls	19.25% Resident: 30.51% staff: 8.26%	August-December, 2005 (120days)	Not identified	NR	• Enhanced cleaning and disinfectants • Universal precautions (with reinforcement of hand hygiene washing) and isolation • Withdrawal of affected workers • Suspension of new admissions and restriction of visitor • Educational workshop	The independent risk factors were age (OR, 5.70; 95% CI 1.53–21.57, in ≥90 years aged person compared to those aged <80 years), floor where the outbreak started (OR, 2.74; 95% CI 1.09–6.86), and cognitive impairment (OR, 2.64; 95% CI 1.04–6.67).

LTCF, long-term care facility; NR, not reported; PPE, personal protective equipment; OR, odds ratio; CI, confidence interval; RR, relative risk; AGE, acute gastro-enteritis; ABHS, alcohol-based hand sanitizer; CDI, *Clostridium difficile* infection; NA, not associated; US, the United States; ILI, influenza like illness; TB, tuberculosis; TST, tuberculin skin test; MRPA, multidrug-resistant *Pseudomonas aeruginosa*; MRSA, methicillin-resistant *Staphylococcus aureus*; HCW, healthcare worker; MDRO, multi-drug resistant organism; HEV, hepatitis E virus; HBV, hepatitis B virus; ER, emergency room.

In total, 37 studies reported 1,332 outbreaks (affecting 1,122 residents and 385 staff members) in 1,182 facilities. There were three prolonged GAS outbreaks of multiple consecutive clusters for over 6 months. The overall attack rates ranged widely from 0.84% to 73.17% in 29 studies. Among the 29 studies, the median of the overall attack rate was 15.73%: 8.27% for bacterial outbreaks and 19.25% for viral outbreaks (Table 3). In 8 studies, it was not possible to calculate the rate due to lack of information. The highest attack rate of 73.17% was reported in an outbreak of respiratory syncytial virus (RSV) and human metapneumovirus (HMPV) [50], followed by *Clostridium difficile* (51.97%) [27] and viral gastroenteritis caused by norovirus and rotavirus (48.6%) [26]. Influenza-like illness had the median overall attack rate of 24.50%. The median attack rate among staff was highest for the acute gastroenteritis outbreaks.

The duration of outbreaks ranged from less than one month to over 6 months. Outbreaks in 13 studies lasted for over 6 months: 3 studies by hepatitis B virus, 3 by GAS, 2 by tuberculosis (TB), 2 by multi-drug resistant organisms (MDROs), 1 by viral gastroenteritis, 1 by *C*. *difficile*, and 1 by hepatitis C virus [15, 17, 19–22, 27, 28, 30, 35, 37, 39, 46].

#### Causes and critical problems contributing to transmission

Causes of transmission in the eligible studies were reported as person-to-person transmission, contaminated water and food, and problems in practice (Tables 2 and 4). The following studies (n = 12) did not report or could not identify the cause of the outbreaks: 5 studies on influenza viruses, 2 on non-typeable *Haemophilus influenzae*, 1 on hepatitis C virus, 1 on *C*. *difficile*, 1 on adenovirus, 1 on norovirus, and 1 on RSV and HMPV [14, 27, 29, 30, 32, 36, 40, 42, 44, 45, 48, 50]. The most commonly reported route of transmission was person-to-person [17, 22–24, 26, 31, 33, 35, 38, 41, 43, 46, 47, 49]. Of these studies, the human source of transmission was identified as the HCWs in six outbreaks [22, 24, 26, 38, 41, 49] and the residents in two outbreaks [17, 33]. The skin of a HCW was a reservoir for Methicillin-resistant *Staphylococcus aureus* (MRSA) outbreaks, leading to cross-infection, in the study by Maltezou et al. [22]. The large gastroenteritis outbreak affecting 394 people in 8 facilities was attributed to staff who worked at multiple facilities [49]. One study showed that three outbreaks of GAS recurred for three years because of continued person-to-person transmission from colonized residents [17]. In the study by Šubelj and Učakar [23], person-to-person transmission resulted from delayed implementation of control measures. Contaminated water and food were sources of infection in five studies [16, 25, 26, 33, 46]. Hepatitis E outbreak was caused by contaminated tap water after heavy rain [25], while the consumption of tap water was the suspected cause of one viral gastroenteritis outbreak [26]. Foodborne causes such as contaminated cake or meals were noted in three studies regarding *Clostridium perfringens*, *Salmonella enteritidis*, and gastroenteritis [16, 33, 46].

Most of the reviewed studies pointed out several issues in practice that might have facilitated the occurrence and spread of outbreaks. The most frequently observed problem was suboptimal hand hygiene, followed by personal protective equipment (PPE), and cleaning and disinfection. Investigation for the GAS outbreak in the study by Nanduri et al. [37] revealed that hand hygiene compliance among employees was 14–25%. Additionally, poor hand hygiene became a more critical factor that facilitated the transmission of acute gastroenteritis, particularly in LTCFs having close living conditions with frequent close contact between the staff and dependent residents [31]. Issues related to PPE had been addressed including inappropriate use of glove and improper storage of PPE [20, 24, 30, 31, 34, 37]. There were reports indicating the potential to cross-contamination by not-changing gloves between residents or by storage of PPE in the room of the index case [31, 34]. Breaches in disinfection and cleaning of the environment and equipment were associated with many outbreaks, most of them were GAS [20, 37, 38] or hepatitis B and C outbreaks [28, 30]. Three reports of GAS outbreaks found lapses in wound care practice such as inconsistent cleaning and disinfection [20, 37, 38]. The outbreaks of MDROs and hepatitis B reported device related issues including sharing of a device and inappropriate use of reusable devices [15, 18, 21, 34]. Hepatitis B and C outbreaks commonly reported that lapses during podiatry care and point-of-care testing procedures (blood glucose test and international normalized ratio monitoring) caused the transmission of bloodborne pathogens among residents [18, 19, 21, 28, 30]. The lapses included the sharing of contaminated equipment, improper disinfection, and poor hand hygiene adherence.

Some studies noted failure of environmental infection control [32, 35, 39, 43]. Two of those studies were TB epidemics, and the investigation revealed that the case residents were exposed to insufficient room ventilation. An influenza outbreak in the summer was facilitated by a heating preventive measure that placed all the residents in one limited area [43]. The response to outbreaks also could influence the progress of outbreaks. Nine reports underlined early notification of outbreaks to public health authorities and implementation of control measures within 3 days of onset of the first case, which affected the attack rates and duration of the outbreaks [23, 29, 35, 41, 44, 46, 48–50].

Some studies on influenza outbreaks discussed issues related to vaccines. Of the three influenza outbreaks in a well-vaccinated population, two studies pointed out that a mismatch between the circulating strains and the vaccine strains affected this population [41, 45], and the other study noted an insufficient vaccine effectiveness [42]. Especially, the study by Burette et al. [41] identified that in addition to the mismatch, several defects including a vaccination rate of 0% among staff and untimely vaccination among residents led to the outbreaks. In addition, they raised the issue of the knowledge and proficiency of general practitioners in influenza diagnosis, treatment, and prevention.

Moreover, three studies suspecting transmission from staff to residents placed emphasis on work restriction of the ill staff [38, 40, 49]. There were other problems including poor personal hygiene of staff members [22], lack of communication between institutions [17], and understaffing [24].

Several studies demonstrated host factors associated with the outbreaks in case-control analysis, that were identified as: age [29], sex [16], cognitive impairment [29], nutritional status [27], comorbidities [23, 24], use of an indwelling device [15, 17], and dependence level [31]. Although not the result of the case-control analysis, the study by Spires et al. [50] reported that all cases of RSV and HMPV were dependent dementia patients, implicating that dependence was an important factor.

#### Control measures

Strategies to control outbreaks were reported in 30 of the 37 reviewed papers, as summarized in Table 5. All 30 studies reported that one or more NPIs were applied to control the outbreaks. From a rigorous perspective, only one study on a multidrug-resistant *Pseudomonas aeruginosa* (MRPA) outbreak implemented all the measures recommended to control an outbreak by pathogens [15]. Work restriction of ill workers was less frequently reported compared to other measures. Only five studies reported the creation of outbreak control teams for effective management of the outbreaks [15, 17, 23, 27, 43]. Most facilities notified public health authorities or institutions about the outbreaks and received advice and assistance to manage the outbreaks. All four studies applying limitation or cessation of group activities were recently published since 2014 [31, 42, 44, 50].

**Table 5:** Control measures applied during outbreaks in the included studies (n = 30*).

Article	Pathogen or illness	Transmission mode	Outbreak control team	Prophylaxis	NPI						
					Standard precaution	Transmission based precaution	Social distancing	Active surveillance	Enhanced training for HCW	Employee work restriction	Environmental control
Luque Fernandez et al. (2008)	Gastroenteritis (Norovirus and Rotavirus)	contact					MI, V				CL
Nguyen et al. (2012)	Norovirus	contact					N	○			
Nicolay et al. (2018)	Norovirus	contact			HH, ○	PPE	MI, G		E, M		DI
Subelj and Ucakar (2015)	Gastroenteritis (Rotavirus)	contact	○		HH	PPE	RU		E	○	DI, CL
Van Esch et al. (2015)	*Clostridium difficile*	contact	○		HH		I	○			DI, CL
Jordan et al. (2015)	Influenza (A)	droplet		R, H(oseltamivir)		D	N, G			○	
Chan et al. (2014)	Influenza (A)	droplet		R(oseltamivir)		RH	I, G	○	M	○	DI
Win et al. (2010)	Influenza (B)	droplet						○			
Bamberg et al. (2010)	Influenza (A)	droplet		R, H(oseltamivir)	HH	D, RH	N, V, RU	○	E	○	
Burette et al. (2009)	Influenza (A)	droplet		R(oseltamivir)			I				
Gaillat et al. (2008)	Influenza (A)	droplet	○	R, H(oseltamivir)		D, C	I				
Khalil et al. (2013)	*Mycobacterium tuberculosis*	airborne					N	○ (contact screening)			
Lai et al. (2016)	*Mycobacterium tuberculosis*	airborne					N, I	○ (contact screening)			EN
Spires et al. (2017)	Respiratory syncytial virus and human metapneumovirus	contact and standard		R(oseltamivir)	HH	D, C, RH	N, V, I, G	○		○	P
Maltezou et al. (2009)	MRSA	contact		R, H		C	MI		E		
Kanayama et al. (2016)	MRPA	contact	○			C, PPE	I, N	○	E		CL, S
Weterings et al. (2015)	KPC-KP	contact			HH	PPE	I, RU	○ (contact screening)	E, M		CL, DI
Dooling et al. (2013)	Group A Streptococcus (GAS)	droplet (and contact)	○	R, H	HH	C		○	E		P, CL, DI
Thigpen et al. (2007)	Group A Streptococcus (GAS)	droplet (and contact)		R, H	○	RH		○			P
Ahmed et al. (2018)	Group A Streptococcus (GAS)	droplet (and contact)				C		○			
Nanduri et al. (2019)	Group A Streptococcus (GAS)	droplet (and contact)		R, H				○			
Kobayashi et al. (2016)	Group A Streptococcus (GAS)	droplet (and contact)				C		○			
Calles et al. (2017)	Hepatitis C virus	blood-borne						○			EN, S, CL, DI
Diercke et al. (2015)	Hepatitis B virus	blood-borne									S
Wise et al. (2012)	Hepatitis B virus	blood-borne		R, H					E, M		S, EN
Sachdeva et al. (2012)	Hepatitis B virus	blood-borne		R			MI	○	E, M		DI, CL, S
Chen et al. (2016)	Hepatitis E virus	fecal-oral					I	○			EN
Van Dort et al. (2007)	*Haemophilus influenzae*	droplet			U	D		○			
Andersson et al. (2015)	*Haemophilus influenzae*	droplet					N	○	E		
Domínguez-Berjón et al. (2007)	Adenovirus (epidemic keratoconjunctivitis)	contact and droplet			HH, U	PPE	I, N, V		E	○	DI, CL

R, resident; H, healthcare worker; HH, (improving) hand hygiene; U, universal precaution; C, contact precaution; RH, respiratory hygiene; D, droplet precaution; PPE, personal protective equipment; I, isolation or cohorting; MI, minimal isolation (non-essential contact restriction or enteric isolation); N, new admission restriction; RU, restriction of transfer between unit; V, visitor restriction; G, minimizing or stopping group activities; E, education; M, monitoring; DI, disinfection; CL, cleaning; P, improving availability or access of product; S, single use of equipment; EN, improving environmental infection control; MRPA, multidrug-resistant *Pseudomonas aeruginosa*; MRSA, methicillin-resistant *Staphylococcus aureus*; KPC-KP, *Klebsiella pneumoniae* carbapenemase-producing *Klebsiella pneumonia*.

*Sena et al. (2013), Blaney et al. (2011), Moffatt et al. (2011), Frank et al. (2007) and Ludwig et al. (2013) were excluded in this table due to not reporting control measure. Barret et al. (2014) and Mahmud et al. (2013) were excluded in this table since they analyzed multiple outbreaks.

#### Gastroenteritis outbreaks (n = 5)

Three studies on gastrointestinal infection, in which adherence to hand hygiene among HCWs was crucial to prevent its spread, reported control measures including stringent hand hygiene practice and reinforcement of standard precautions [23, 27, 31]. Only two studies implemented barrier precautions by use of PPE [23, 31]. All five studies that reported control measures used various types of social distancing measures including isolation, restriction of new admission and visitors, or cessation of group activities [23, 26, 27, 31, 49]. Active surveillance by symptom reporting for early detection of new cases was reported in two studies [27, 49]. Although four studies reported that staff members were affected by the outbreaks, only one study implemented work exclusion for ill employees and showed the lowest attack rate among staff [23]. Four studies reported intensive cleaning and disinfection of the environment [23, 26, 27, 31]. The implementation of more stringent procedures for cleaning and disinfection with diluted bleach was reported for outbreaks of *C*. *difficile* [27]. The study by Luque et al. [26] on viral gastroenteritis reported a relatively small number of interventions, showing a high attack rate of 48.63%. On the other hand, the study by Šubelj and Učakar [23] with the largest number of control measures among the five studies had a lower attack rate of 11.21% compared to the other outbreaks.

#### Influenza virus outbreaks (n = 6)

Five of the six reports implemented prophylactic oseltamivir for the residents and/or HCWs [40–44]. Both droplet precaution and active surveillance were reported in 3 of the six studies. Five of the studies on influenza outbreaks reported a total of 172 cases among staff, but only three of the studies implemented the measure of work restriction [40, 42, 44]. The study by Burette et al. [41] reported the lowest number of control measures including prophylaxis and isolation and had the highest attack rate of 42.05% among the five reports on influenza A.

#### Tuberculosis outbreaks (n = 2)

Following the detection of the index case, two reports on tuberculosis outbreaks conducted case finding among residents and staff by contact tracing [35, 39]. Responding to the outbreaks, measures for the cases included isolation and transfer to a hospital in one study [39], but the other study only restricted new admissions [35]. Neither of them mentioned airborne precautions taken such as N95 respirators. Investigations in both reports found that the air exchange rates of the rooms were inadequate. The study by Lai et al. [39] corrected the failure of the environmental infection control by increasing the ventilation rates in the building. Both outbreaks involved cases among workers, but there was no description about the work status of the affected staff after the occurrence.

#### MDROs outbreaks (n = 3)

Three outbreaks of MDROs were caused by MRSA, MRPA, or *Klebsiella pneumoniae* carbapenemase-producing *Klebsiella pneumonia* (KPC-KP) [15, 22, 34]. The MRSA outbreak study applied mupirocin eradication for the residents and staff [22]. All three outbreaks used transmission-based precautions and quarantine measures to prevent the spread of MDROs. In addition, they all provided re-education for the staff to improve infection control practice. Furthermore, two of the reports on MRPA and KPC-KP intensified the cleaning of the environment to interrupt the contamination of the environment. The study by Kanayama et al. [15] demonstrated that the sharing of a device such as a suction device was linked with the MRPA cases; thus, the control measures included stopping the sharing of devices.

The unexpected occurrence of the KPC-KP cases led to contact surveillance for additional exposure cases [34]. The investigation for the KPC-KP outbreak revealed poor hand hygiene compliance among staff; thus, interventions including frequent audit and feedback were implemented.

#### GAS outbreaks (n = 5)

Three of the five GAS outbreaks provided antibiotic prophylaxis to the residents and staff [17, 24, 37]. All five studies conducted surveillance culture for active case finding. None of the five outbreaks reported droplet precautions, but the study by Thigpen et al. [24] mentioned an enhanced respiratory hygiene practice. Although three of the GAS outbreaks lasted for a long period due to an unsolved person-to-person transmission [17, 20, 37], none of the studies implemented social distancing measures. Two studies improved the availability of hand dispensers to address the suboptimal hand hygiene practice that was revealed during their observation [17, 24]. None of the studies on the three outbreaks involving sick employees reported encouragement of work exclusion for ill staff [20, 37, 38], but some studies reported that there were voluntary sick leaves of employees before the recognition of the outbreaks.

#### Hepatitis virus outbreaks (n = 5)

Prophylaxis of hepatitis B vaccine and immuno-globulin were implemented for hepatitis B virus outbreaks in two studies [21, 28]. There is not much generally recommended NPIs for the hepatitis B and C outbreaks; thus, the studies on these outbreaks reported fewer NPIs than those on the other outbreaks. Three of the studies on the hepatitis virus outbreaks tried to find additional cases by serologic screening [21, 25, 30]. All the studies on the hepatitis B and C virus outbreaks employed the principle of single-use device or individual equipment to break the chain of infection [18, 21, 28, 30]. Improvement of the care room was done in two studies that found lapses in the environment of the procedure room [28, 30]. Interventions for drinking water standards and toilets were reported in the hepatitis E virus outbreak caused by contaminated water [25].

#### Heamophilus influenzae outbreaks (n = 2)

One of the two *H*. *influenzae* outbreaks reported droplet precaution during the outbreak [32], and the other study restricted new admissions to prevent additional transmission [36].

#### Other outbreaks (n = 2)

The study on the RSV and HMPV outbreak reported various measures including active surveillance, isolation, contact precaution, antiviral prophylaxis for residents and work restriction for ill staff to control respiratory pathogen transmission [50].

In the epidemic keratoconjunctivitis outbreak, control measures involved universal precaution with enhanced hand hygiene, isolation and restriction of visitors, and work restriction for affected workers [29].

## Discussion

We updated the understanding of outbreaks in LTCFs with more recently published reports. This review also explored and summarized critical issues facilitating the spread of the outbreaks and the control measures, which have not been addressed in detail in the previous review [9]. Lessons learned from the results of this review would enable better prevention and control of outbreaks in LTCFs in the future. Implications and suggestions for achieving the best response to epidemics by LTCFs, and for future research concerning outbreaks, have been described in this review.

The most common outbreaks in LTCFs in this review were respiratory infections followed by GI infections, showing consistency with the findings of a previous study on nursing homes [51]. Interestingly, there is a difference in the outbreak reports for MDROs compared to the previous review [9]. This review identified three reports including MRSA, MRPA, and KPC-KP, suggesting the increase of multidrug-resistant organisms, given that the prior review found only two reports of MRSA [9]. As the prevalence of MDROs is increasing in LTCFs [2], they become a particular concern in these facilities. With drug resistance on the rise, MDRO related outbreaks may occur in LTCFs with growing frequency. It shows that staff and managers of LTCFs need to be aware of the significance of this trend and to prepare a plan.

Influenza viruses and GAS accounted for a large number of single pathogens. This is similar to the results of the previous review showing that the largest number of aetiologic agents affecting outbreaks was influenza viruses in LTCFs from 1966 to 2008 [9]. First, in this review, five of the six influenza virus outbreaks occurred by the influenza A virus and the other by influenza B virus. Influenza-like illness included in the studies showed a median attack rate of 24.50%, similar to that of seasonal influenza, usually 20–30% [40]. Vaccine-related issues have been raised in influenza outbreaks that have occurred in LTCFs of highly immunized residents. This finding suggests some implications to prevent influenza outbreaks in LTCFs. Regarding vaccination coverage among staff, the study by Thomas [52] found that influenza episodes were reduced if an employee was vaccinated, and the Centers for Disease Control and Prevention (CDC) recommend that all healthcare worker get vaccinated annually [53]. Therefore, influenza vaccination among healthcare personnel should be considered to mitigate the risk of influenza outbreaks in LTCFs. Additionally, because vaccination does not provide complete protection, active daily surveillance for influenza-like illness is still recommended for all persons in LTCFs during influenza season [53], which is evident by the outbreaks occurring in highly immunized LTCF population. Secondly, the GAS infection rate among older adults in LTCFs is from 3 to 8-fold higher than that of community-dwelling older adults, due to risk factors such as grouped living conditions, and underlying diseases [54, 55]. Five of the studies on the GAS outbreaks in this review reported a median attack rate of 2.43% in LTCFs, which is within the range of 1–30% reported in the previous study [56]. Three of them were long-lasting outbreaks with multiple clusters for more than 6 months, which suggested that an accurate identification of how pathogens spread was a fundamental step in outbreak control.

This review also explored critical issues on practices that propagated the occurrence and spread of outbreaks. Consequently, failure to adhere to basic infection control practice, including hand hygiene, disinfection, and cleaning, was found to be a practical issue of great importance on the transmission of the outbreaks in LTCFs. Some reports even mentioned that this issue ultimately caused their outbreaks [19, 21, 28]. Most studies showed that this problem contributed to their outbreaks by causing cross-contamination between hands, environments, and equipment. First, the hands of HCWs may be the sources of the outbreaks. Frequent close contacts between residents and HCWs in LTCFs increase the risk of widespread outbreaks. Incorrect hand hygiene among HCWs can result in hands remaining contaminated, and this may lead to the transfer of organisms to the environment and to other residents [57]. Like previous studies that already confirmed poor compliance with hand hygiene among HCWs [4], one of the included studies reported that hand hygiene compliance was less than 30% [37]. Semmelweis demonstrated the role of hand hygiene in preventing infections transmitted by person-to-person [58]. Hand hygiene has a significant effect on reducing GI and respiratory infections [59]. The WHO recommends that hand hygiene should be performed at the following 5 key moments: before and after touching a patient, before clean/aseptic procedures, after body fluid exposure risk, and after touching a patient’s surroundings [57]. Promotion of hand hygiene compliance through multimodal strategies has been proven to reduce healthcare-associated infection [60]. Multifaceted interventions such as WHO-5 strategies (including system change, training and education, monitoring and feedback, reminder and communication, and culture of safety) are generally effective in increasing and sustaining hand hygiene compliance at various settings [61–65]. The same evidence has been reported from studies on LTCFs, suggesting improved hand hygiene reduces the infection rate or respiratory outbreaks [66, 67].

Secondly, lapses in cleaning and disinfection could make equipment and the environment become a reservoir for transferring pathogens [68]. Most of the studies regarding this issue were on outbreaks of GAS, gastroenteritis, and hepatitis B and C virus, and they found a failure to adhere to proper disinfection and cleaning principles. First of all, the outbreaks of GAS and hepatitis B and C were linked with breaches in specific procedures. GAS outbreaks were usually relevant to wound care and hepatitis B and C to point-of-care testing. With the aforementioned hand hygiene, disinfection and cleaning were basic infection control practices that are included in standard precautions. The standard precautions consist of hand hygiene, environmental cleaning, reprocessing and disinfection of care equipment, waste and linen management, the prevention of needle stick injuries, and the use of personal protective equipment (PPE), if necessary [11]. The practice of standard precautions is the imperative basic approach for IPC that was applied to all residents assuming they had the potential for pathogen transmission [11]. Standard precautions are necessary practice, especially in LTCFs where the systems for diagnostic tests are poor and active surveillance is not generally done. Tailored ongoing education with multimodal strategies for HCWs would ensure that basic infection control principles and standard precautions are integrated into daily practice such as point-of-care testing [69]. As a result, a reduction in threats of outbreaks can be guaranteed, as well as the safety and health of persons residing or working in LTCFs. Meanwhile, the studies on the outbreaks of gastroenteritis reported that there were lapses in decontaminating environment. Environmental contamination may have a critical role in the spread of these outbreaks. Importantly, norovirus and *C*. *difficile* that are capable of surviving in the environment for long periods of time require more consideration in environmental disinfection [70, 71]. For norovirus, the CDC has recommended more frequent cleaning and disinfection of rooms and high-touch surfaces with a hypo-chlorite (1000–5000 ppm) or other proper disinfectant [72]. The most effective control method for *C*. *difficile* was reported as disinfection and cleaning of rooms and high-touch surfaces with a chlorine-based solution (5000 ppm) [70, 71].

This systematic review identified control measures taken during the outbreaks, especially non-pharmaceutical interventions. The results showed that the actual application of control measures may be far from what is recommended and implied that there are several challenges to overcome in outbreak management at LTCFs. First, acute care facilities like hospitals can successfully manage outbreaks through collaborative efforts with multiple experts [73]. However, most of the LTCFs in this review requested advice from public health authorities and organizations for unexpected outbreaks instead of organizing a multidisciplinary team. This may imply that LTCFs do not have sufficient capacity and expertise to individually plan, implement, and evaluate the management of outbreaks. Forming a local support network between acute hospitals and LTCFs at a regional level would be a potential way to close the gaps and to enhance outbreak control practices in LTCFs without adequate capacity [74]. Furthermore, training infection control professionals in facilities could facilitate early detection of outbreaks and timely interventions.

Secondly, this review found that many LTCF employees were affected by the outbreaks, which is consistent with the finding of the previous review [9]. However, it also revealed that work restriction for ill staff was not implemented well during the outbreaks in the LTCFs, which was not reviewed in the prior review [9]. Gastroenteritis outbreaks in this review had a higher median attack rate among staff than the other outbreaks, but only one study among them reported the application of work exclusion. Moreover, there were some reports that implied transmission attributable to sick employees. These results pointed out the role of presenteeism in LTCFs. Presenteeism among sick employees may have a role in either introducing pathogens or facilitating the transmission of outbreaks. The CDC recommends work restrictions for health care workers infected with or exposed to diseases such as diarrheal diseases, GAS, tuberculosis, and viral respiratory infection [75]. However, it may be challenging for LTCFs with fewer available resources to implement the exclusion of ill staff during outbreaks, given the fact that one study reported difficulty from understaffing as a result of work restriction [50]. The study by Widera et al. [76] suggested that daily screening of all staff members for symptoms during outbreaks on every shift may mitigate the impact of presenteeism. Considering that presenteeism is associated with various factors such as job security and lack of paid sick leave [77], further discussion is needed for plans addressing this issue in LTCFs.

Lastly, additional challenges in managing the outbreaks in LTCFs were reported. They included understaffing, insufficient supply of products such as PPE, lack of expertise, and limited application of isolation [41, 46, 47, 50]. Many long-term care facilities have difficulty in applying isolation of infected persons due to the limited availability of isolation rooms [5] and concern about the adverse effects of isolation and additional precautions may affect the compliance with related practices [78]. For the same reason, some studies in this review used minimal types of isolation like enteric isolation. If single rooms are not available, facilities should consider applying the cohort measure or bed curtains as another method of isolation. In regard to this challenge, a study by Dumyati et al. [73] suggested a shift towards enhanced standard precautions or risk-based application of transmission-based precautions to uphold the quality of life of residents by HCWs. Future research should identify the rationale for the safety and effectiveness of this strategy or other options. Additionally, one qualitative study found that misunderstanding of the key concepts and recommendations of IPC contributed to under-utilization of transmission-based precautions [78]. Thus, emphasis should be on training and education of HCWs on transmission-based precautions.

A majority of the reviewed studies assessed infection control practice as part of the investigation to identify the problem areas of the outbreaks. Most studies attempted on-site direct observation of infection control practices and product availability. Some studies of retrospective design used survey and interview among employees. However, it is difficult to find a study that investigated the compliance rate of control measures during the outbreaks. Only a few studies described gaps in the actual application after recommendations for control measures were made. Even several studies overlooked reporting control measures, especially NPIs [3]. Although this does not necessarily mean that they did not apply measures, for the purpose of this review some studies were excluded from the analysis of control measures. The Outbreak Reports and Intervention studies of Nosocomial Infection (ORION) statement was developed and recommended, to improve the quality of outbreak reporting [79]. According to the statement, control measures should be included in the paper. Future studies should consider following the ORION statement for reporting of outbreaks [79], which would facilitate the formation of a body of evidence for outbreak management in LTCFs.

This study has several limitations. First, we only included studies written in English. It is possible that our review missed articles of interest written in other languages. Second, we conducted only a qualitative review due to the variability of the outbreak reports. Third, the quality assessment was conducted on studies of certain design including case-control and cohort study due to applicability of the quality assessment tool. Fourth, the results of this review have limited generalizability due to publication bias, given that either successfully controlled outbreaks or outbreaks with higher attack rates or fatality rates tend to be published.

## Conclusions

This update for understanding outbreaks in LTCFs by reviewing recent studies indicates that staff members and residents are still at risk for contagious disease outbreaks including influenza, gastroenteritis, and GAS infection. As for the problem aspects, rather than by new or unexpected issues, violation of basic infection control practices was found to facilitate the occurrence and onward transmission of pathogens. The results of this review suggest that LTCFs need to inspect basic infection control practice and to implement them thoroughly in daily care as priorities. Efforts should be directed to promoting consistent and optimal adherence to the basic practice of infection control among HCWs at all times in LTCFs.

When an outbreak occurs, non-pharmaceutical control measures should be utilized to interrupt transmission. However, work restriction was infrequently taken compared to other measures. Given the fact that over half of the included studies reported at least one employee ill and their possible role in the spread of pathogens, it is necessary that symptomatic staff members temporarily preclude themselves from working. In addition to work restriction, though, LTCFs with poor resources have faced various challenges in outbreak management. Further discussion and studies are needed to identify the way addressing these challenges.

## Supporting information

S1 File PRISMA checklist. (PDF)
